# Rheological Properties and Kinetics of Gelation of Binary Polymers between Xanthan Gum and Locust Bean Gum

**DOI:** 10.3390/polym15234604

**Published:** 2023-12-02

**Authors:** Hui Zhang, Zhun Yan, Fan Xie, Yanjun Tian, Lianzhong Ai

**Affiliations:** Shanghai Engineering Research Center of Food Microbiology, School of Health Science and Engineering, University of Shanghai for Science and Technology, Shanghai 200093, China; zhh8672@126.com (H.Z.);

**Keywords:** xanthan gum, locust bean gum, synergistic interaction, gelling property, kinetics of gelation

## Abstract

The synergistic interaction and gelling kinetics between xanthan gum (XG) and locust bean gum (LBG) at different mass ratios (XG/LBG 9:1, 7:3, 5:5, 3:7, 1:9) were investigated using a rheometer. The results showed that the mixtures of XG and LBG induced gel formation, and the strongest gel structure was found for the mixture of XG/LBG 3:7 according to the yield stress, storage modulus (G′), and power law parameters. Temperature ramp studies indicated that heating destroyed the gels at 55~60 °C, while cooling induced the sol–gel transition at around 52 °C for all mixtures. Structure developing rate (SDR) curves showed that XG/LBG 3:7 exhibited the highest SDR during the cooling ramp among all the samples. Non-isothermal kinetic analysis demonstrated that the gelation process of XG/LBG mixtures during cooling included two steps: a high-temperature region (55~39 °C) needing higher activation energy (*Ea*, 111.97 to 199.20 kJ/mol for different mixtures) and a low-temperature region (39~20 °C) needing lower *Ea* (74.33 to 85.31 kJ/mol), which indicated higher energy barriers to overcome at the initial stage of gel formation. The lowest *Ea* of 74.33 kJ/mol was found for XG/LBG 3:7 in the low-temperature region. Scanning electron microscopy (SEM) showed that the gel of XG/LBG 3:7 presented the densest entanglements. These results indicated the strongest synergism interaction occurred in XG/LBG 3:7 to form gel network structures. This study will help promote the application of XG-LBG blends to design novel food structures.

## 1. Introduction

Polysaccharide-based hydrocolloids are commonly applied in the food industry due to their thickening, gelling, and stabilizing properties [1]. However, they are always used in combination with more than two types of polysaccharides to achieve the desired functions. This is because synergistic polysaccharide–polysaccharide interactions enhance or change the functions of the individual polysaccharides, thus drastically expanding their applications [2]. Such synergistic interactions are commercially attractive as they may impart economic benefits and be used to generate novel functionality or manipulate texture and rheology with reduced amounts of gum [3,4].

Xanthan gum (XG) is an exopolysaccharide produced by *Xanthomonas* bacteria. It comprises glucose, mannose, and glucuronic acid in a molar ratio of 2:2:1 with varying proportions of *O*-acetyl and pyruvyl groups [5]. Due to its superior rheological properties, like high viscosity and pseudo-plasticity in solutions, XG has been extensively used in a wide range of applications, from the food industry to oil drilling [6]. However, the rheological properties of XG are related to temperature, resulting in conformational transitions from helix to random coil structure. In addition, individual XG solutions cannot form 3D network structures like starch [7] and konjac glucomannan [8], which are needed to exhibit their gelling properties.

Locust bean gum (LBG) is a typical galactomannan with an average mannose-to-galactose ratio of about 3.5 [9]. Similar to XG, LBG can also form viscous aqueous solutions at relatively low concentrations and is able to stabilize emulsions and replace fat in many food products [10]. In addition, LBG can synergistically interact with XG to form a thermoreversible gel with more elasticity and strength, which is an obvious advantage to both hydrocolloids in terms of their use in industrial applications [11].

Actually, the synergistic interaction between XG and LBG in aqueous solutions has been studied for several decades [12]. The mass ratio of XG to LBG plays an important role in the synergistic interaction. Tako, Asato, and Nakamura [13] reported maximum dynamic modulus with the mixing ratio of XG to LBG at 1:2, and the interaction mechanism was proposed to be a lock-and-key effect between the side chains of XG and the backbone of LBG. Higiro, Herald, and Alavi [14] reported that the 60% XG and 40% LBG mixture exhibited the strongest attraction due to a more flexible conformation formed in the XG-LBG complex. In addition, the contents of the acetate and pyruvate groups in XG were also reported to be involved in the interactions [15,16]. A recent study further found that the ordered XG interacted with LBG through side chains, while the disordered XG and LBG formed gels by backbone–backbone interactions [17]. The interaction mechanisms and blending conditions of XG and LBG needed to form gels are being discussed in this paper. However, the rheological features of the gels in food systems and the kinetics of the gelation of binary polymers between XG and LBG are still unclear.

In this study, binary mixtures of XG and LBG at certain ratios were prepared to simulate gelling in foods; the rheological properties of the mixtures, including oscillation amplitude tests, oscillation frequency sweeps, and temperature ramps, were studied. The rate of gel formation and the kinetics of the sol–gel transition were analyzed. The morphological features of the gels were observed using a scanning electron microscope (SEM). This work aimed to explore the gel features of XG-LBG blends and develop their applications to design novel food structures.

## 2. Materials and Methods

### 2.1. Materials

Xanthan gum (XG, 80) was purchased from CP Kelco (Rizhao, Shandong, China) Biological Co., Ltd., and locust bean gum (LBG, 646) was provided by Danisco (Shanghai, China) Co., Ltd. The average molecular weights (Mw) of XG and LBG were measured as 4171.0 and 1054.0 KDa, respectively, by high-performance size-exclusion chromatography coupled with a multi-angle laser light-scattering detector (HPSEC-MALLS). All other reagents used were of analytical grade, unless otherwise specified.

### 2.2. Sample Preparation

The XG and LBG powders were mixed according to the mass ratios of 9:1, 7:3, 5:5, 3:7, and 1:9. The corresponding mixtures were suspended in Milli-Q water at a concentration of 1% (m/V) and heated at 80 °C for 2 h with continuous stirring in sealed tubes to completely solubilize the gums. The obtained solutions were then cooled and left overnight at room temperature.

### 2.3. Rheological Characterizations

Rheological measurements of XG/LBG mixtures and the individual dispersions were performed using a DHR-3 hybrid rheometer (TA instrument, New Castle, DE, USA) with a steel flat plate (40 mm). The samples were first heated to 80 °C to become fluids, and then loaded onto the plate carefully with a gap distance of 1 mm. The temperature was controlled using a circulating water bath coupled with a Peltier temperature control device. The geometry-containing sample was covered with low-viscosity silicon oil and a sample hood to prevent water evaporation during testing. Before all rheological measurements, samples were cooled to 25 °C and maintained on the plate for 600 s to allow temperature equilibrium.

Oscillation amplitude tests were conducted at 25 °C with an increasing strain from 0.1 to 1000.0% and frequency of 1 Hz. Oscillation frequency sweeps were tested in a range of 0.1–10.0 Hz with a strain of 5%. Temperature ramps were performed by heating samples from 20 to 80 °C at a rate of 5 °C/min, holding at 80 °C for 10 min, and subsequent cooling from 80 to 20 °C at a rate of 5 °C/min at a frequency of 1 Hz and strain of 1%. TRIOS software (Version 3.3.0.4055) was employed for data collection and analysis.

### 2.4. Determination of Structure Development Rate

The structure development rate (SDR) during cooling was applied to deduce the change in storage modulus during the gel formation process, defined as (*dG′*/*dt*) and calculated by first fitting fifth-order polynomials to the *G′*-time data recorded by the rheometer.

SDR(*t*) = *dG′*(*t*)/*dt* (1)

where SDR(*t*) is the gel formation rate at the time *t* (Pa·s^−1^); *G′*(*t*) is the storage modulus (*G′*) at the time *t* (Pa).

### 2.5. Scanning Electron Microscope Observation

Scanning electron microscopy (SEM) was used to investigate the microstructures of different gel samples. The gel samples were put into a specimen holder and immersed in liquid nitrogen (−207 °C) for freeze drying. The dried samples were coated with gold and transferred to the SEM for observation (Hitachi S-570 SEM, Hitachi Lts., Tokyo, Japan). Different parts of each sample were detected at 10 kV at different magnifications.

### 2.6. Statistical Analysis

The rheological data were collected and treated by TA instruments Trios software (Version 3.3.0.4055) and Origin 8.0. All the measurements were conducted in triplicate and expressed as means ± standard deviation (SD), which were then subjected to analysis of variance (ANOVA), with *p* < 0.05 indicating statistical significance.

## 3. Results

### 3.1. Oscillation Amplitude Tests

**Figure 1:**
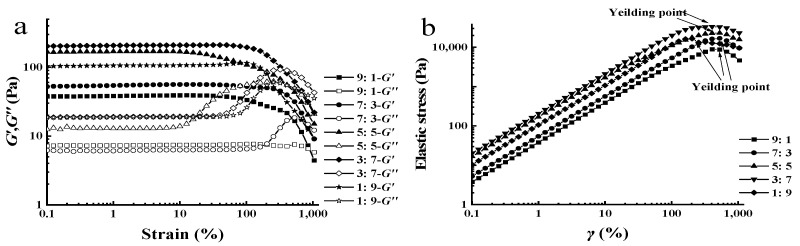
Strain dependence (**a**) and yield stress points (**b**) of dynamic moduli of XG/LBG mixtures at different mass ratios (1.0%, m/V).

Strain sweep tests were carried out to identify the linear viscoelastic region (LVR) of the blended XG-LBG–systems, as shown in Figure 1a. The results show that both the storage modulus (*G′*) and loss modulus (*G″*) of these mixtures at different mass ratios of XG to LBG were plateaus, and *G′* > *G″* when the strain amplitude was less than 10%. These results demonstrated that all the samples had wide LVRs and that strong physical entanglements existed in XG/LBG blends, making them resistant to mechanical deformation [18]. However, the entanglements could be destroyed as the strain increased to more than 10%. The XG/LBG 3:7 mixture possessed the highest *G′* and *G″*, indicating enhanced mechanical strength. In addition, strain overshoot behaviors (SOBs), as displayed in *G″*, were found in the blends at the XG/LBG ratios of 7:3, 5:5, 3:7, and 1:9. It is believed that the phenomenon of SOB is caused by the formation of structured complexes between XG and LBG [19,20].

The dynamic strain sweep data can be used to obtain the elastic stress σ (σ = *G′* × γ) and then plotted as a function of the strain amplitude. The maximum in the elastic stress versus strain curve is interpreted as the yield stress [21]. Yield stress is an important parameter to evaluate the internal network structure of polymers. It represents the minimum shear stress required to initiate fluid flow, which is capable of predicting the processing of the bolus in the mouth [18].

Figure 1b shows the elastic stress versus strain of XG/LBG mixtures at a polymer concentration of 1.0%. The results demonstrated that all the yield stresses of XG/LBG mixtures were significantly higher than those for XG alone at the same concentration, as reported previously [18,19]. The lowest yield stress was observed for the mixture of XG/LBG 9:1 (8.85 × 10^3^ Pa), while the highest yield stress was found for the mixture of XG/LBG 3:7 (3.33 × 10^4^ Pa). These results indicated that stronger three-dimensional structures were formed when XG was mixed with LBG. The highest yield stress for the mixture of XG/LBG 3:7 implies that much higher shear stress is needed to overcome the remarkable structure.

### 3.2. Oscillation Frequency Sweeps

**Figure 2:**
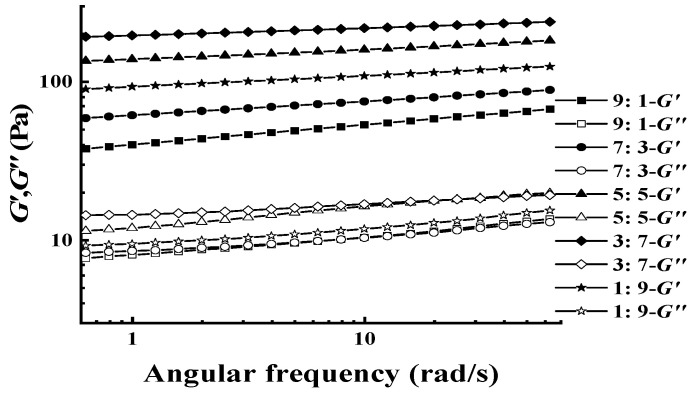
Plots of *G′* and *G″* versus angle frequency of different XG/LBG mixtures at different mass ratios (1.0%, m/V).

The dynamic viscoelastic properties (frequency dependence of *G′* and *G″*) of different XG/LBG blends were investigated by oscillation frequency sweeps within the linear viscoelastic region (strain of 5%) at 25 °C, and the results are shown in Figure 2. All the profiles of *G′* exceeded those of *G″* with negligible frequency dependence on both moduli over the whole experimental range, suggesting that all the mixtures showed typical gel-like behaviors.

Complex modulus G^*^ ($G^{*}=\sqrt{{G^{\prime }}^{2}+{G^{\prime \prime }}^{2}}$) is used to evaluate the gel strength and the total resistance to deformation of a material considered to be an elastic solid [22]. When *G′* is much higher than *G″*, G^*^ can be considered to be equal to *G′*. In the current study, the values of *G′* of all the samples were more than ten times *G″*; therefore, the parameter of *G′* can be used instead of G^*^ to evaluate the gel strength of XG/LBG complexes. As shown in Figure 2, the values of *G′* increased with the increase in the LBG ratio in the XG/LBG mixtures, and the highest *G′* was found in the mixture of XG/LBG 3:7. However, *G′* decreased when the amount of LBG was further increased (XG/LBG 1:9). This indicates that the optimal synergistic effect of XG and LBG occurs at a ratio of 3:7. Other studies have also reported that increasing the amount of LBG in XG can induce the formation of gel [11,23].

The power law relationship between *G′* and *ω* was used to evaluate the gel strength using the following equation:

(2) $$G^{\prime }=a\times \omega^{b}\;$$

where *G′* is the storage modulus, *ω* represents the oscillation frequency (rad/s), *a* is a constant, and *b* is an exponent that can be obtained as the slope in a double logarithmic plot of *G′* versus *ω*. It is known that the *b* value is related to the strength and nature of gels. For true gels, log *G′* versus log *ω* plots have a zero slope (*b* = 0); for weak gels, such plots have positive slopes (*b* > 0), and *G′* has a higher magnitude than *G″* [24]. Generally, low *b* values are characteristics of elastic gels, while *b* values near 1 indicate that the system behaves as a viscous gel [25].

Table 1 shows the fitting parameters derived from the power law equation of XG-LBG mixtures at different mass ratios. The results indicated that the *b* values for all the samples were low (0.05~0.13) and that the higher content of LBG in mixtures led to lower *b* values in all the samples except for XG/LBG 1:9. This implies that LBG supplemented into XG could induce strong physical gels. In addition, the constants of *a* increased gradually as the XG/LBG mass ratio decreased from 9:1 to 3:7, indicating that stronger elastic structures are formed as LBG is compounded into XG. The lowest *b* value and highest *a* value for XG/LBG 3:7 indicated the strongest gel strength and elastic structure [25].

### 3.3. Temperature Ramp Tests

**Figure 3:**
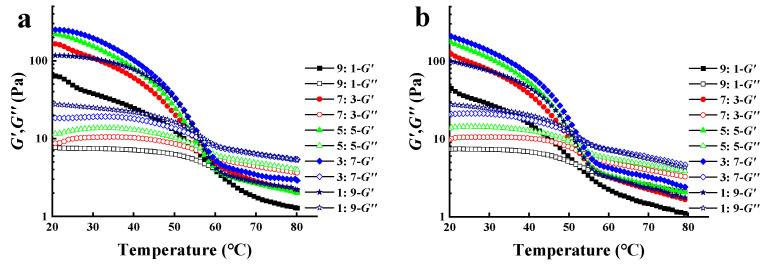
Plots of *G′* and *G″* versus temperature of different XG/LBG mixtures of different mass ratios (1.0%, m/V); (**a**) shows the curves for the heating ramp and (**b**) shows the curves for the cooling ramp.

The effects of temperature on the gelling properties of XG/LBG mixtures were studied by conducting temperature ramp tests. Figure 3 shows the changes in *G′* and *G″* during heating (Figure 3a) and cooling (Figure 3b) between 20 and 80 °C for different XG/LBG gels (1% *w*/*v*). As shown in Figure 3a, both *G′* and *G″* of different XG/LBG gels decreased as temperature increased, and *G′* decreased more rapidly than *G″*. This might be due to the enhancement of molecular motion during heating, which weakened the non-covalent bonding between XG and LBG [26]. The gel–sol transition of biopolymers occurred at the temperature where *G′* = *G″* (gel melting temperature point, *T*_gel-sol_) [27]. From Figure 3a, the crossover points of *G′* and *G″* were found at 50~60 °C for all samples, meaning that the gel of the XG/LBG mixtures melted during the heating process and that the gum behavior transitioned from a solid-like state to a solution. The *T*_gel-sol_ decreased from 60 °C (XG/LBG 9:1) to 55 °C (XG/LBG 1:9) with a decreasing XG amount in the blends. This indicates that XG may be the predominant contributor to maintaining gel structure during heating. It was reported that the decrease in *G′* was mainly due to the helix–coil transition of XG, which strongly affects the whole solution rheology [16]. The double-stranded helix of XG is destroyed as the temperature increases, thus providing fewer lock-and-key positions for the XG/LBG interaction and weakening the synergy at high temperatures [28].

Figure 3b shows the change in the modulus (*G′* and *G″*) during the cooling process from 80 to 20 °C. The results indicate that both *G′* and *G″* increased slowly as the temperature decreased from 80 to 60 °C, but that the samples were still in a solution state as *G′* < *G″*. As the temperature continued decreasing, both *G′* and *G″* increased rapidly, and a sol–gel transition point (*T*_sol-gel_) was found for each sample where *G′* = *G″*, which were 50.2, 52.0, 52.7, 53.7, and 52.8 °C for XG/LBG 9:1, 7:3, 5:5, 3:7, and 1:9, respectively. The gel structures were formed after the temperatures dropped below *T*_sol-gel_. Gel formation could be due to the renaturation of xanthan conformation, from disorder to helix order, as the temperature decreases [29,30]. The lower *T*_sol-gel_ compared to *T*_gel-sol_ observed in the different samples was ascribed to the thermal inertia of the measuring system [16].

### 3.4. Structure Developing Rate (SDR) Analysis during Cooling

**Figure 4:**
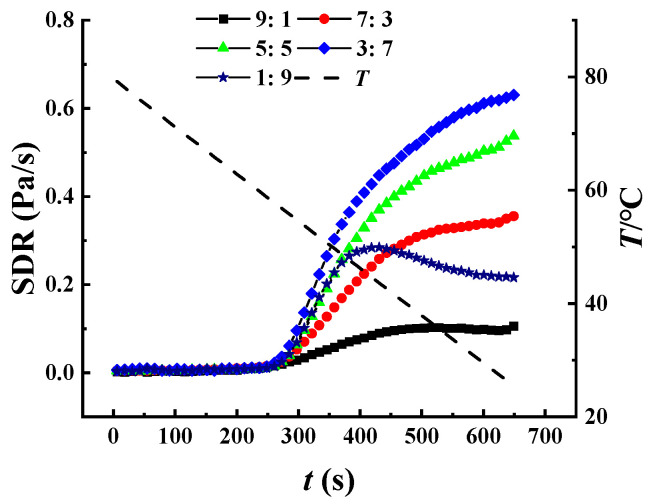
Structure development rate (SDR) of different XG/LBG mixtures at different mass ratios (1.0%, m/V) during cooling process.

The first derivation, dG′/dt, was used for the calculation of the structure developing rate (SDR) of XG/LBG mixtures, which is commonly used to evaluate the structural changes in gels [31,32]. Figure 4 shows the SDR of different XG-LBG mixed systems during cooling. The results showed that the SDR of the mixed systems was low, with no significant increase in G′ when temperatures decreased from 80 to 55 °C. As the temperatures gradually decreased to 20 °C, the SDR values of all samples increased significantly, indicating that the gel structures were preliminarily forming in the system. The sigmoidal shape of the SDR curves, as shown in Figure 4, is a typical feature for the formation of ordered structures in biopolymers [27,33]. Therefore, it can be concluded that the loose random coiled conformation of XG gradually transformed into an ordered helix structure when the temperature reached the conformational transition temperature, resulting in tight interactions with LBG and leading to a rapid increase in gelation rate.

Among all the XG/LBG mixed systems, the sample of XG/LBG 3:7 possessed the highest SDR value, while XG/LBG 9:1 exhibited the lowest, indicating that the former had the fastest formation rate of gel network structure. This result is consistent with the oscillation frequency sweeps, showing that the strongest synergistic interaction occurred when the mass ratio of XG to LBG was 3:7. As for the sample of XG/LBG 1:9, the SDR value initially increased slowly (80~55 °C), then rapidly increased to its peak (55~40 °C), and then decreased again (40~20 °C). This might be due to XG occupying a smaller proportion in this system and the limited formation of helix structures as the temperature decreased. When the temperature reached 40 °C, the maximum number of helix structures was formed by XG, and thus, the peak SDR value was achieved. As the temperature continued to decrease beyond that point, the SDR value no longer increased.

### 3.5. Non-Isothermal Kinetics Analysis for Gel Formation

Determining the kinetics for gel structure development during the cooling of biopolymers is difficult due to the combined effects of time and temperature. In this study, a non-isothermal kinetic model was adopted to analyze the sol-gel transition of XG/LBG mixtures while linearly decreasing the temperature at a constant rate [34,35]. The non-isothermal kinetic model is based on a combination of the reaction rate, the Arrhenius equation, and the time–temperature relationship as follows:

(3) $$\mathrm{Ln}\left(\frac{1}{G^{\prime n}}\times \frac{dG^{\prime }}{dt}\right)=\ln k_{0}-\frac{E_{a}}{RT}$$

where *G′* is the storage modulus, *n* is the order of reaction rate, *t* is the reaction time (s), *k_0_* is the Arrhenius frequency factor, *E_a_* is the activation energy of the process (J/mol), *R* is the universal gas constant (8.314 J/mol∙K), and *T* is the absolute temperature at reaction time *t* (K). According to Wu, Hamann, and Foegeding [36] and Da Silva, Gonçalves, and Rao [37], macromolecular aggregation can be described as a bimolecular association equilibrium with an assumed reaction order of *n* = 2.

**Figure 5:**
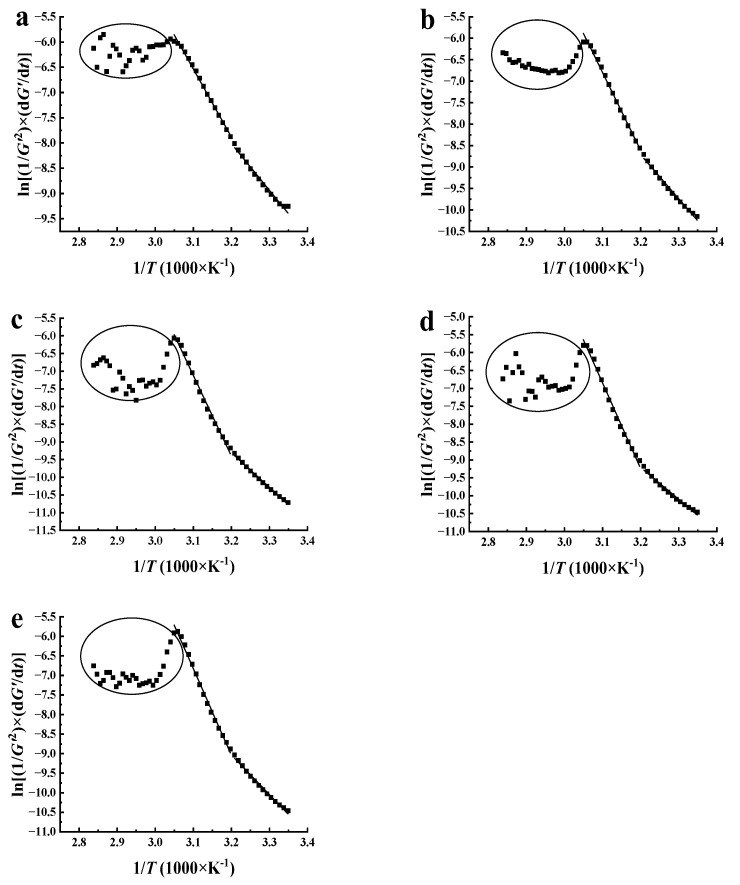
Arrhenius fitting curve of *G′* with temperature for XG/LBG mixed systems; (**a**–**e**) are the curves for the samples of XG/LBG 9:1, 7:3, 5:5, 3:7, and 1:9, respectively.

Arrhenius plots of the different mixed systems were obtained by plotting $ln(1/[{G^{\prime }}^{n}]\times dG^{\prime }/dt)$ against 1/T, and the activation energy of the gelling process was estimated using a linear least-squares routine (Figure 5). As shown in Figure 5, the model was prone to unstable fluctuations when the temperature was higher than 55 °C, resulting in noise signals appearing on the fitting curve (as shown in the elliptical part of the figure). This might be because the ordered structure of XG had not yet formed, and interactions between XG and LBG were unstable. As the temperature continued to decrease, the ordered structure of XG recovered, and the interaction between XG and LBG was enhanced. The gelling process can be satisfactorily described by a two-step process corresponding to two different temperature ranges. The slopes of the curves in the high-temperature range (55~39 °C) are steeper than those in the low-temperature range (39~20 °C), indicating that higher energy is needed to overcome the intermolecular interaction between XG and LBG in the high-temperature region. The activation energy *E_a_* of different samples was estimated with fitting coefficients of 0.981 to 0.994, as shown in Table 2. It can be seen that the *E_a_* values of all samples in the high-temperature region were between 111.97 and 199.20 kJ/mol, significantly higher than those in the low-temperature region (74.33 and 85.31 kJ/mol). This suggests that the molecular motion intensified and that a higher energy barrier was required for the development of gel network structures at high temperatures. Conversely, molecular chains are more prone to aggregation, with lower energy barriers to network structure formation at low temperatures. A similar phenomenon was also found in other biopolymers, as reported previously [37,38].

For the sample of XG/LBG 3:7, *E_a_* reached its maximum value (199.20 kJ/mol) at a higher-temperature region than all the mixed systems. Since the *E_a_* value can be considered the energy barrier for the gelation process, the highest *E_a_* value for XG/LBG 3:7 has the most difficulty in the gelation. Obviously, this is contradictory to the results of dynamic viscoelastic rheology. It is believed that the initial preparation for gelation is essentially caused by hydrogen bonding and involves short chain segments as well as conformational changes from a random coil to an ordered helix of xanthan strands [39]. So, the high *E_a_* value for XG/LBG 3:7 might be due to the high energy barrier to the initial preparation for gelation in the high-temperature region. However, the lowest *Ea* value for XG/LBG 3:7 (74.33 kJ/mol) in the low-temperature region suggests strong synergism between XG and LBG to form lateral aggregates and junction zones via hydrophobic interaction [40].

### 3.6. Microstructure of XG/LBG Gels

**Figure 6:**
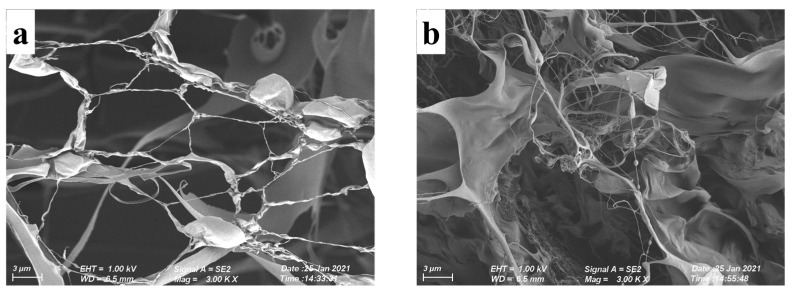

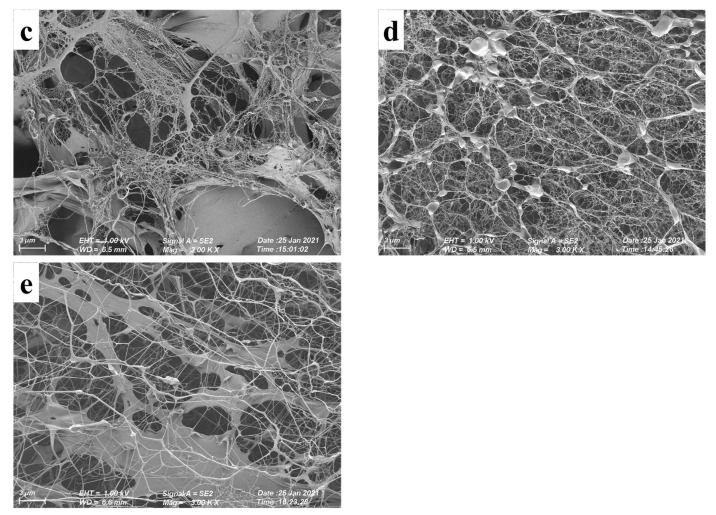
Scanning electron micrographs of gel structure of different XG/LBG mixtures at different mass ratios (magnification: 3.0 K, scale bar = 5 μm); (**a**–**e**) are the images for the samples of XG/LBG 9:1, 7:3, 5:5, 3:7, and 1:9, respectively.

SEM was applied to observe the microstructure of different XG/LBG gels. As shown in Figure 6, the images present obviously different gel networks for the XG/LBG complexes at different mass ratios. The sample of XG/LBG 9:1 showed a sparse network. As the mass ratio of LBG increased, the entanglements between mixed polymers increased, and the networks became more and more compact. The sample of XG/LBG 3:7 presented the densest entanglements, indicating the strongest interaction between XG and LBG to form gel network structures. These results are consistent with the aforementioned rheological studies.

## 4. Conclusions

In conclusion, the interaction between XG and LBG could induce the gelling property of their mixture, and the strongest interaction and gel strength were found for the mixture of XG/LBG 3:7. Heating or cooling induced the gel–sol or sol–gel transition of XG/LBG mixtures, which might be due to the helix–coil transition of XG as the temperature changed. During the cooling process, XG/LBG 3:7 showed the highest SDR, the fastest formation rate of gel network structure, and the densest gel structure. During the initial preparation of gelation during cooling, XG/LBG 3:7 needed to overcome the highest energy barrier. However, as the temperature decreased to 39 °C, the lowest *Ea* value was found for XG/LBG 3:7, which confirmed the strongest synergism at this mass ratio. This study demonstrated how a fine-tuning of the properties of the mixed gels was possible through the variation of mass ratios of two polymers, which helps provide strategies for the application of XG-LBG blends to design novel food structures.

## Figures and Tables

**Table 1:** Power law parameters of storage modulus and oscillation frequency for XG-LBG mixtures.

Mass Ratio of XG/LBG	$G^{\prime }=a\times \omega^{b}$		
	*a*	*b*	R^2^
9:1	1.59 ± 0.02 ^e^	0.13 ± 0.00 ^a^	0.995
7:3	1.79 ± 0.01 ^d^	0.09 ± 0.00 ^b^	0.998
5:5	2.15 ± 0.01 ^b^	0.06 ± 0.00 ^d^	0.999
3:7	2.29 ± 0.00 ^a^	0.05 ± 0.00 ^e^	0.999
1:9	1.99 ± 0.02 ^c^	0.07 ± 0.00 ^c^	0.999

Different letters in the same row indicate significant differences at *p* < 0.05.

**Table 2:** Activation energy for sol–gel transition process and correlation coefficient of XG/LBG mixed systems.

Mass Ratio of XG/LBG (*w*/*w*)	High-Temperature Region (55~39 °C)		Low-Temperature Region (39~20 °C)	
	*Ea*/(kJ/mol)	R^2^	*Ea*/(kJ/mol)	R^2^
9:1	111.97	0.994	76.93	0.981
7:3	150.67	0.993	85.31	0.987
5:5	189.56	0.991	81.98	0.994
3:7	199.20	0.991	74.33	0.981
1:9	184.07	0.992	84.55	0.988

## Data Availability

The data presented in this study are available on request from the corresponding author.
